# Retirement Preparedness: How Important Is Being Financially Literate?

**DOI:** 10.1093/geroni/igaa057.1440

**Published:** 2020-12-16

**Authors:** Mengya Wang, Suzanne Bartholomae

**Affiliations:** 1 Iowa State University, ames, Iowa, United States; 2 Iowa State University, Ames, Iowa, United States

## Abstract

Financial security in retirement is a major concern for many Americans. Numerous studies document that Americans are not prepared for retirement, with financial illiteracy cited as one reason Americans fail to plan. Employing data from the 2018 National Financial Capability Study (N=27,091), this study investigates actual financial literacy (AFL) and perceived financial literacy (PFL) and how combinations of this measure influences retirement planning, and varies based on years from retirement. This study found relatively low financial literacy and retirement preparedness levels among the US sample, even for those pre-retirees ages 55 to 64. Individually, PFL and AFL increased as one approached retirement. When combined, adults nearing retirement (55 to 64) comprised the greatest proportion of the high AFL and high PFL (29.9%) group compared to adults 20 years or more from retirement (18-44) who largely made up the low AFL and PFL (48%) group. Based on a logistic regression, adults closest to retirement (ages 55 to 64) are more likely to be planning compared to the other groups, as are adults who were financially confident, risk takers, highly educated, males, and white. Compared to adults with high AFL and high PFL, adults with low AFL and low PFL, or a combination (low PFL and high AFL, high PFL and low AFL) have lower odds of preparing for retirement. Both PFL and AFL influences retirement planning, and PFL may be as important as AFL. Our highlight the importance of policies and programs to support Americans with retirement planning.

